# Tamoxifen-associated vasculitis in a breast cancer patient

**DOI:** 10.1186/1477-7819-5-9

**Published:** 2007-01-23

**Authors:** Myrna Candelaria, Rafael Hurtado-Monroy, Pablo Vargas-Viveros, Silvia Carrillo-Muñnoz, Alfonso Duenas-Gonzalez

**Affiliations:** 1 Department of Hematología Oncología, Hospital Angeles del Pedregal. Mexico city, Mexico; 2 Division of Research. Instituto Nacional de Cancerología. Mexico city, Mexico; 3 Unidad de Investigaciones Biomédicas en Cáncer. Universidad Nacional Autónoma de México, Mexico

## Abstract

**Background:**

Estrogen plays a critical role in breast cancer. Thereafter, endocrine therapy is a standard of care in patients with breast carcinoma, expressing ER or PR.

**Case presentation:**

Herein we report the case of a 53-year old patient, who developed cholestasis and vasculitis during the treatment with tamoxifen. This toxicity was reversable after the removal of the drug. Thereafter she continued adjuvant treatment for breast carcinoma with anastrazole. Since tamoxifen has been widely indicated for patients with breast carcinoma, we did a literature review, looking for other cases with this type of toxicity.

**Conclusion:**

This case is the third with vasculitis informed in the literature, but the first one that additionally developed cholestasis and arthritis. Although it is rare, we discuss the indication of this drug in the actual era, where aromatase inhibitors offer a better security profile.

## Background

Breast cancer is the most common cause of cancer death in women worldwide. Rates vary about fivefold around the world, but they are increasing even in regions that until recently had low rates of disease [1] Endocrine treatment is indicated in hormone-sensitive patients. Tamoxifen is an oral antiestrogen, first used in metastatic breast cancer in the early 1970s. Large clinical trials were initiated in the late 1970s and early 1980s to test the drug's role as adjuvant therapy in early stage breast cancer. Observations of marked decreases in the development of contralateral breast cancer among tamoxifen recipients suggested potential for the drug in chemoprevention of breast cancer. The most recent analysis of the Early Breast Cancer Trialists' Collaborative Group (EBCTCG) which included information on 37,000 women in 55 trials of adjuvant tamoxifen, published in 2005, confirms the value of this oral antiestrogen. Among women with ER-positive disease, the reduction in the recurrence rate and in the breast cancer death rate are highly significant either after 1–2-year or 5-year use of tamoxifen, but are greater in the latter. This indirect evidence that 1–2 years is less effective than 5 years of tamoxifen in ER-positive disease is highly significant for recurrence, and for breast cancer mortality, and is supported by the directly randomised comparisons of different tamoxifen durations. In patients with ER-negative disease, tamoxifen may or not produce some benefit either after 1–2 years or 5 years of tamoxifen. For women with tumors of unknown ER status the benefits are slightly weaker than those for women with ER-positive disease [2].

In regard to its chemopreventive properties, a number of prevention trials with tamoxifen have shown a 48% reduction in ER-positive cancers [3]. Based on these findings, the United States Preventive Services Task Force recommended women with risk factors for breast cancer should discuss the potential benefits and harms of tamoxifen with their doctors [4].

The use of adjuvant tamoxifen has been associated with certain toxic effects. The most important is the development of endometrial cancer that occurs at a rate that is 2 to 7 times greater than that observed in untreated women [5,6]. Tamoxifen is also associated with an increased incidence of deep venous thrombosis, pulmonary emboli and stroke [7-11]. Other less frequent encountered side effects are benign ovarian cysts, [12] and ophthalmologic alterations [13]. Toxic effects of short-term toxic effects of tamoxifen may include vasomotor and gynecologic symptoms such as vaginal discharge or irritation [14]. A case of vasculitis induced by tamoxifen was reported in 1990 [15] and since then no other cases are reported in literature.

## Case presentation

A 53-year old woman was admitted to emergency room with one week history of purpuric lesions limited to both legs, accompanied by paresthesias, very painful arthralgias which confined her to wheel chair and distal edema in both lower extremities. She had a previous history of modified mastectomy with axillary lymphadenectomy for a retroareolar, canalicular breast carcinoma 6 months before, whose histological diagnosis was a ductal carcinoma measuring 4.5 cm of diameter, estrogen and progesterone receptor-positive and HER2 negative. She received adjuvant radiation and started tamoxifen 20 mg daily four months before admission. At the clinical examination, she had several purpuric papular lesions in both distal extremities, some were confluent, within a 1 cm diameter on the left external heel. Arthritis was also present in both ankles and knees. Liver function tests demonstrated a cholestatic damage, with increase of alkaline phosphatase (196; normal: 40–150 U/L), GGT (213; normal> 12–43), SGPT (249; normal 5–66 U/L), SGOT (110; normal 9–55 U/L); other liver tests, and the rest of laboratory, including SMA-18, blood cytology, coagulation studies, viral hepatitis infection markers, antinuclear antibodies, coagulation tests and crioaglutinines were normal. Tumor markers, including CA15-3 were negative or normal. Liver ultrasound showed none focal lesions and was considered normal. Histopathology analysis of skin biopsies showed deposits of fibrinoid material in the walls and infiltration by lymphocytes and neutrophils in dermis vessels, which was diagnosed as vasculitis.

Tamoxifen was withdrawn and methylprednisolone at 1 g daily was administered during 3 days. Thereafter, 0.5 mg/kg prednisone was indicated during 7 days and subsequently decreased. Lesions disappeared after one week. Currently she is alive and well receiving anastrazole.

## Discussion

Tamoxifen is a widely used drug in the treatment of breast cancer. Endometrial cancer and thrombosis-related phenomena are well-known side-effects of this drug, however, because of its ample use it is important to be aware of other uncommon toxicities. Although skin changes caused by tamoxifen were reported in 19% of tamoxifen treated patients [15], and also published medical literatureidentified occasional reports of skin reactions (urticaria) in patients receiving tamoxifen [16-18], the development of purpuric vasculitis due to tamoxifen is extremely unsual, since only two cases of purpuric vasculits have been reported in the literature [19,20]. In the first case [19], also a mild elevation of aspartate aminotransferase level was documented, but neither a clear cholestatic damage or arthritis; a clear drug-relation was documented, since vasculitis disappeared after tamoxifen withdrawal, and reappeared when this drug was newly indicated. The second case [20] was recently published, and describes the dermal vasculopathic changes in a patient who required tamoxifen, after progression with fulvestrant, but, as the first one, none other clinical manifestations, including liver function tests abnormalities or arthritis were present. The authors concluded that the quick resolution of this toxicity after tamoxifen discontinuation strongly supported a cuase-effect in this patient. A comprehensive list of the cases reported in literature to date is detailed in Table 1.

**Table 1:** Tamoxifen associated vasculitis. Case reports in the literature.

Reference	Age	Duration of tamoxifen intake	Other tamoxifen related toxicities/Comments
19	56	6 months	Mild increase of liver enzymes: AST (65; normal <40 U/L), and mildy decrease C4 (10; normal 15–45 mg%). Skin lesions reappeared when tamoxifen was newly indicated
20	67 y	4 weeks	None other toxicity was documented. Tamoxifen was withdrawn.
This case	53 y	4 months	Increase of liver enzymes: alkaline phosphatase (196; normal: 40–150 U/L), GGT (213; normal> 12–43), ALT (249; normal 5–66 U/L), AST (110; normal 9–55 U/L). Very painful arthritis in knees, ankles

Other severe toxicities, such as liver toxicity, thromboembolism and endometrial carcinoma have also been described [5,6,8,10,21]. Among drug-induced cholestasis, estrogens, anabolic steroids and structurally similar congeners such as tamoxifen are well-described causes of this syndrome [21], whereas acute inflammatory arthritis has only been reported in three cases [22]. The cases of acute inflammatory polyarthritis in association with tamoxifen had none liver damage, and also neither skin lession. Our case report is unique in the sense that the patient showed the association of purpuric vasculitis, cholestasis and arthritis. Tamoxifen, a non-steroidal antiestrogen, has been used in the treatment of breast cancer since the early 1970s. Tamoxifen binds to estrogen receptor (ER) and inhibits estradiol binding to ER, resulting in decreased tumor cell proliferation and cell death. In addition to antitumor responses, tamoxifen can also exert antiangiogenic effects. According with the Stockholm trial [23], tamoxifen was considered the standard of care for adjuvant treatment in node-negative postmenopausal patients, with improvement of DFS and overall survival. Thereafter the NSABP B-14 trial [24] confirmed this results and informed a 12% absolute difference in DFS, a 28% reduction in treatment failure, and 22% reduction in mortality after 5 years of treatment. This nonsteroidal estrogen antagonist was the only approved adjuvant treatment of hormone positive breast carcinoma. However, initial results from the ATAC trial [25] demonstrated a clearly benefit of the use of anastrazole in such patients, with prolongation of the time to recurrence, a 58% reduction of primary contralateral breast cancers, as well as, increase of DFS. A longer follow-up of these patients [26], confirmed the benefit of this aromatase inhibitor. Other aromatase inhibitors have also been evaluated in this setting: letrozole, administered as an extended therapy, after 5 years-tamoxifen, showed a lower risk of recurrence by 43% and improvement of the estimated 4-year DFS by 6% and OS by 2% [27]. The Intergroup Exemestane Study also demonstrated that the addition of exemestane (as sequential therapy) after tamoxifen increased the DFS to 91.5%, although the OS was not modified. These results have demonstrated a clearly benefit of aromatase inhibitors, compared with tamoxifen, as adjuvant treatment for breast carcinoma, and also a better sequrity profile, since a significant reduction in hot flushes, vaginal discharge, vaginal bleeding, ishcemic cerebrovascular events, and endometrial cancer has been informed. On the other hand, these drugs exhibit more musculoskeletal disorders, osteoporosis and fractures. On basis of the important efficay, with a better sequrity profile, they should be considereded as the first line of adjuvant treatment in patients with breast carcinoma, in particular anastrazol, which is the only drug approved by the FDA in the adjuvant setting, without a previous tamoxifen therapy, that may avoid severe side effects, as occurred in this patient.

## Conflict of interest

The author(s) declare that they have no competing interests.

## Authors' contributions

MC: Conceived and wrote the manuscript and treated the patient.

RH-M: treated the patient and reviewed the manuscript.

PV: Treated the patient and searched for literature.

SC: Searched for literature and reviewed the manuscript.

AD_G: reviewed critically the manuscript.
